# Exploratory case series of circulating tumor DNA dynamics during tandem therapy in metastatic castration-resistant prostate cancer

**DOI:** 10.1186/s13550-026-01388-x

**Published:** 2026-02-11

**Authors:** Mariam Amghar, Tobias Rausch, Hilal Özgür, Mareike Roscher, Ulrike Bauder-Wüst, Frank Bruchertseifer, Alfred Morgenstern, Vladimír Beneš, Clemens Kratochwil, Martina Benešová-Schäfer

**Affiliations:** 1 Junior Research Group Translational Radiotheranostics, German Cancer Research Center (DKFZ), Im Neuenheimer Feld 280, 69120 Heidelberg, Germany; 2 Genomics Core Facility, European Molecular Biology Laboratory (EMBL), Heidelberg, Germany; 3 Service Unit Radiopharmaceuticals and Preclinical Studies, German Cancer Research Center (DKFZ), Im Neuenheimer Feld 280, 69120 Heidelberg, Germany; 4 European Commission, Joint Research Centre (JRC), Karlsruhe, Germany; 5 Department of Nuclear Medicine, University Hospital Heidelberg (UKHD), Heidelberg, Germany

**Keywords:** Prostate cancer, PSMA, Targeted radionuclide therapy, Tandem actinium-lutetium therapy, ctDNA

## Abstract

**Background:**

Prostate-specific membrane antigen (PSMA)-targeted radiopharmaceutical therapy (RPT) with the alpha-emitter actinium-225 (^225^Ac) has shown promising activity in metastatic castration-resistant prostate cancer (mCRPC), but its use is limited by toxicity. A tandem approach combining [^225^Ac]Ac-PSMA-617 and [^177^Lu]Lu-PSMA-617 (actinium-lutetium) has been developed to mitigate adverse effects and optimize efficacy. Given the scarcity of ^225^Ac and the emergence of resistance, early identification of non-responders is crucial. Circulating tumor DNA (ctDNA), especially tumor fraction (TFx) estimated from ultra-low-pass whole genome sequencing (ULP-WGS) using ichorCNA, may provide a non-invasive biomarker for monitoring treatment response. Blood samples were collected from mCRPC patients treated with actinium-lutetium bimonthly. Cell-free DNA (cfDNA) was extracted and analyzed by ULP-WGS (≤ 7 × coverage). TFx was derived using ichorCNA and compared longitudinally with prostate-specific antigen (PSA) and imaging data.

**Results:**

Patient 1 achieved complete remission with undetectable PSA and TFx after two cycles. Patient 2 showed a strong and sustained therapeutic response; however, after a prolonged treatment hiatus, ctDNA analysis detected genomic progression despite stable PSA levels. Patient 3 initially responded to therapy, but TFx began rising during the third cycle, preceding PSA relapse, indicating early disease progression. Patient 4, classified as a non-responder, showed only transient TFx reduction and experienced rapid disease progression, with increasing PSA and TFx levels already by the second cycle.

**Conclusions:**

Longitudinal ctDNA TFx dynamics enable early detection of response and resistance to actinium-lutetium in mCRPC and may complement PSMA imaging to guide timely, personalized treatment decisions.

**Graphical abstract:**

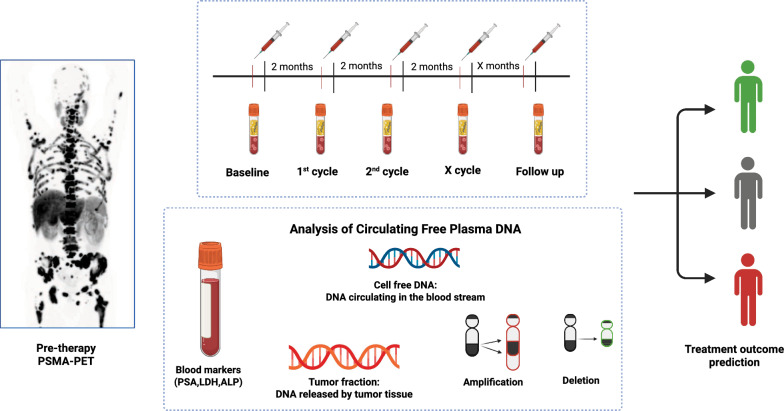

**Supplementary Information:**

The online version contains supplementary material available at 10.1186/s13550-026-01388-x.

## Introduction

In 2022, [^177^Lu]Lu-PSMA-617 (Pluvicto^®^) marked a significant turning point in prostate cancer therapy, gaining Food and Drug Administration and European Medicines Agency approval due to its pronounced clinical efficacy in heavily pre-treated patients [1, 2]. Despite these achievements, approximately 30% of metastatic castration-resistant prostate cancer (mCRPC) patients eventually fail to respond to beta radiation, owing to inherent or acquired resistance [3]. Pioneering clinical trials have explored the combination of PSMA-617 with the alpha-emitting radionuclide ^225^Ac ([^225^Ac]Ac-PSMA-617), demonstrating remarkable efficacy in heavily pre-treated mCRPC patients with diffuse bone marrow infiltration who no longer respond to conventional therapies, including Pluvicto^®^ [4, 5]. [^225^Ac]Ac-PSMA-617 is highly effective against microscopic metastatic disease, leveraging ^225^Ac short tissue range (50–100 µm) [5, 6]. Its radiobiologic properties, while potent for localized tumor ablation, present challenges in treating larger tumor masses and are associated with significant toxicities, particularly higher-grade xerostomia, complicating its use as a standalone therapy. Over time, various radiotherapeutic strategies have been evaluated, with tandem therapy emerging as a means to optimize efficacy and minimize adverse effects [4, 7, 8]. Despite the clinical success of tandem [^225^Ac]Ac-/[^177^Lu]Lu-PSMA-617 (actinium-lutetium) therapy, which leverages the complementary benefits of both radionuclides, identifying and addressing resistance mechanisms remains a significant challenge in treating mCRPC [4]. Current clinical management of mCRPC often relies on standard biomarkers, such as prostate-specific antigen (PSA), lactate dehydrogenase (LDH), and alkaline phosphatase (ALP) [9], along with imaging techniques that may not provide timely insights into treatment efficacy or emerging resistance. Currently, [^68^Ga]Ga-PSMA-11 (Illuccix^®^, Locametz^®^ and Gozellix™), [^18^F]DCFPyL (PYLARIFY^®^ and PYLCLARI^®^), [^18^F]PSMA-1007 (Radelumin^®^), and [^18^F]rhPSMA-7.3 (Posluma^®^) are the Food and Drug Administration-approved gold standards for prostate cancer imaging, enhancing biopsy precision and therapeutic monitoring significantly [10], with other PSMA radiotracers also being used depending on the study site [11]. The scarcity of ^225^Ac, high treatment costs, and xerostomia underscore the urgent need to prioritize early identification of patients who benefit the most from PSMA-radiopharmaceutical therapy (PSMA-RPT) and to discontinue treatment once the patient acquires resistance [12–14]. In this context, non-invasive liquid biopsies, particularly circulating tumor DNA (ctDNA), have emerged as a promising tool for early detection of clinical outcomes, overcoming the limitations of traditional circulating tumor markers by being agnostic to cancer type [15]. Circulating free DNA (cfDNA) refers to fragments of DNA released into the bloodstream, originating from cellular processes such as apoptosis and necrosis. In cancer patients, a subset of cfDNA originates from tumor cells, known as ctDNA. Analysing ctDNA provides a non-invasive method to monitor tumor dynamics, offering insights into genetic alterations, tumor burden, and treatment response. A key metric in this analysis is the tumor fraction (TFx), which represents the proportion of ctDNA within the total cfDNA pool [16, 17]. Assessing TFx is critical for evaluating tumor progression and therapeutic efficacy has been already explored in numerous studies [18–21]. Advanced bioinformatic tools, such as ichorCNA, allow precise estimation of TFx by detecting copy number alterations (CNA)–gains and losses of genomic DNA characteristic of cancer–through ultra-low-pass whole genome sequencing (ULP-WGS) of cfDNA [22]. The aim of this study is to leverage ichorCNA to analyse dynamic ctDNA profiles across treatment cycles in mCRPC patients undergoing actinium-lutetium tandem therapy. Specifically, we seek to investigate possible associations between ctDNA profiles and clinical outcomes.

## Material and methods

### Patients

**Fig. 1:**
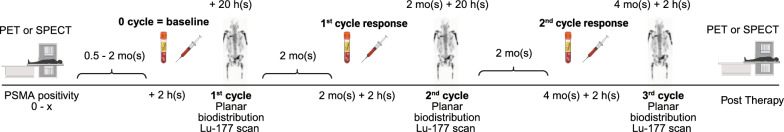
Schematic overview of study workflow and sample collection. Prior to treatment initiation, patients underwent PSMA assessment by PET or SPECT to confirm eligibility for therapy. Before each treatment cycle, blood samples were collected for cfDNA extraction and PSA measurement. Therapy administration was followed by planar whole-body scintigraphy, and post-therapy response evaluation was performed using PET or SPECT imaging

[^225^Ac]Ac-/[^177^Lu]Lu-PSMA-617 was administered as an alternative treatment in adherence to paragraph 37, "Unproven Interventions in Clinical Practice," of the revised Declaration of Helsinki and consistent with German medical guidelines [23]. Enrolment required patients to provide informed consent, including approval from the ethics committee of University Hospital Heidelberg (S-882/2020). The administered activities ranged from 1.5 to 7.4 GBq for [^177^Lu]Lu-PSMA-617 and 4 to 6 MBq for [^225^Ac]Ac-PSMA-617. The rationale for the treatment regimen has already been described previously [24]. Prior to therapy initiation, routine laboratory tests, including complete blood count, renal and hepatic function, and standard biomarkers (PSA, LDH, ALP), were performed to monitor patient status. Blood samples for ctDNA analysis were obtained simultaneously during these routine assessments. All patients were selected based on PSMA-positive lesions detected via [^99m^Tc]Tc-PSMA-GCK01 for single photon emission computed tomography (SPECT) as well as [^18^F]PSMA-1007 for positron emission tomography (PET). All patients were naïve to PSMA-RPT, indicating no prior exposure to radionuclides before undergoing actinium-lutetium treatment. A schematic representation of the samples and imaging acquisition are represented in Fig. 1. Each patient had received comprehensive pre-treatment regimes as detailed in Table 1.

**Table 1:** Baseline patient clinical characteristics and metastatic involvement who received [^225^Ac]Ac-/[^177^Lu]Lu-PSMA-617

	Hormonal therapy	Surgery	Chemotherapy	Metastatic sites	Radiation type
Patient 1	ADT, Enzalutamide, Olaparib	Radical prostatectomy	2 C Docetaxel/Carboplatin 6 C Cisplatin/Etopoxid	PUL, OSS, HEP, LYM	External radiation
Patient 2	ADT, Enzalutamide, Prednisone, Abiraterone, Olaparib	Radical prostatectomy Lymphadectomy	6 C Docetaxel 2 C Cabazitaxel	OSS	External radiation
Patient 3	Leuproreline, Bicalutamide, Enzalutamide	No	2 C Docetaxel	OSS, MAR, HEP	Palliative external radiation
Patient 4	Enzalutamide, Abiraterone/Prednisone	Radical prostatectomy	12 C Docetaxel 6 C Cabazitaxel	OSS, MAR, HEP	Not applied

ADT: Androgen deprivation therapy, C: cycle, HEP: Hepatic, LYM: Lymph nodes, MAR: Bone marrow, OSS: Osseous, PUL: Pulmonary

The table includes data on hormonal therapy, surgical interventions, chemotherapy cycles, metastatic spread, and radiation treatments

### Sample collection and processing

Venous blood samples (10–30 mL) were prospectively collected in EDTA-coated tubes (BD Biosciences, Franklin Lakes, New Jersey, USA) and processed within 60 min of collection upon the patients’ arrival at the clinic, prior scheduled therapy. Once collected, the clinical team notified the research team, and samples were transported from the Nuclear Medicine Department of the University Hospital Heidelberg to the DKFZ for processing. The samples underwent density gradient centrifugation at 2000 × g for 10 min at 4 °C without braking to separate the plasma and buffy coat employing Heraeus Christ Minifuge GL 4400 centrifuge (Heraeus Christ, Osterode am Harz, Germany). The plasma was carefully isolated, transferred to 15 mL Falcon tubes, and separated from the buffy coat. Immediately after processing, the plasma samples were frozen at –80 °C to ensure preservation until further analysis. cfDNA was extracted from plasma using QIAamp MinElute ccfDNA-Midi-Kit (Qiagen, Hagen, Germany). The quantification was determined with Qubit dsDNAHS-Assay-Kit (Thermo Fisher, Karlsruhe, Germany) using Qubit 4 Fluorometer (Thermo Fisher, Karlsruhe, Germany; Q33238). cfDNA library preparation was carried out by the Collibri PS DNA Library Prep Kit (Thermo Fisher, Karlsruhe, Germany) for Illumina sequencing platforms. A total of 10–20 ng of cfDNA was used as input for ULP-WGS library preparation. The input volume was adjusted according to cfDNA stock concentration; in most cases ≥ 20 µL was used, with volumes up to 25 µL permissible, in accordance with the manufacturer’s protocol. Sequencing was performed in paired-end mode (2 × 100 bp) to the requested depths using a NextSeq sequencing instrument. Each cfDNA sample was subjected to ULP-WGS, with sequencing coverage reported in Supplementary Table 1.

### IchorCNA

Genome-wide copy number profiles and TFx were estimated from ULP-WGS data using the ichorCNA algorithm (https://github.com/broadinstitute/ichorCNA) in R (version 3.3.1). ichorCNA uses a probabilistic model, to simultaneously segment the genome, predict large-scale CNA, and estimate the tumor fraction of a cfDNA sample [22].

## Results

This study presents a broad spectrum of patient responses to tandem actinium-lutetium therapy, encompassing examples of complete remission, partial response, mixed response, and non-response. This study includes four patients with a median age of 69.5 years (range: 63–86 years). The clinical pre-treatment characteristics and initial diagnoses of the patients are detailed in Table 1 and Supplementary Table 2. Below, we provide a detailed overview of each patient’s treatment course and outcomes with a focus on TFx and established biomarkers as marker for treatment response.

### Patient 1 – complete remission

**Fig. 2:**
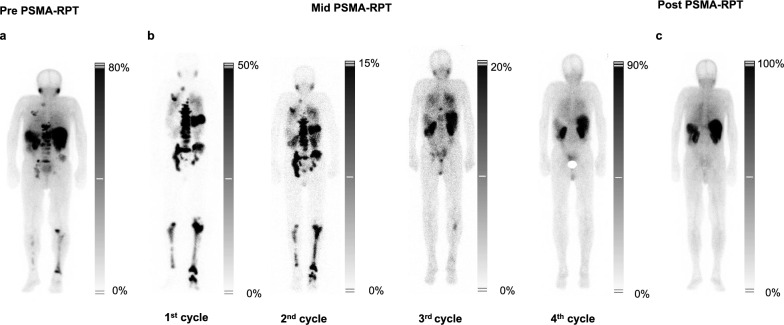
(**a**) Pre-, (**b**) mid-, and (**c**) post-treatment PSMA-SPECT with [^99m^Tc]Tc-PSMA-GCK01 for Patient 1, treated with [^225^Ac]Ac-/[^177^Lu]Lu-PSMA-617

Patient 1, with widespread metastatic involvement identified on PSMA-SPECT using [^99m^Tc]Tc-PSMA-GCK01 (Fig. 2A), underwent actinium-lutetium therapy which led to a dramatic reduction in PSA levels, from 0.50 ng/mL to a nadir of < 0.01 ng/mL, a –98% drop after two cycles (Supplementary Fig. 1.A). Simultaneously, TFx decreased from 0.47 to below detection levels (Supplementary Fig. 1.B), as reflected in CNA profiles (Supplementary Fig. 2). These improvements were mirrored in imaging data as well as tumor biomarkers LDH and ALP, both of which showed a marked decline (Supplementary Fig. 1.C, 1.D). Post-treatment PSMA-SPECT imaging demonstrated complete remission (Fig. 2C), with magnetic resonance imaging confirming eradication of liver metastases (Supplementary Fig. 3.C). Renal function and pre-existing anaemia remained stable, though episodic post-treatment leukopenia were observed (Supplementary Fig. 4). Overall, Patient 1 exhibited a robust response to therapy, characterized by the complete eradication of PSMA-positive metastases.

### Patient 2 – partial responder

**Fig. 3:**
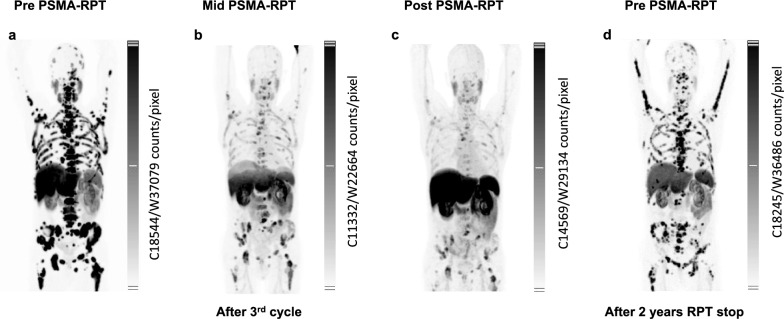
(**a-c**) [^18^F]PSMA-1007 PSMA-PET overviews of Patient 2 treated with [^225^Ac]Ac-/[^177^Lu]Lu-PSMA-617. (**d**) PSMA-PET imaging with [^18^F]PSMA-1007 performed following a two-year treatment break and a therapy switch to Olaparib and Abiraterone

Patient 2, showing PSMA-positive metastases during [^18^F]PSMA-1007-PET (Fig. 3A), exhibited a PSA decline from 190 ng/mL to 21.2 ng/mL, –88.8% over four cycles of actinium-lutetium therapy (Supplementary Fig. 1.E), despite the persistence of bone metastases (Fig. 3C). This reduction in PSA was accompanied by a dramatic decrease in TFx, dropping from an initial value of 0.46 to below detection levels after the first cycle and remaining undetectable throughout the subsequent three cycles (Supplementary Fig. 1.F, Supplementary Fig. 5). After reaching the cumulative renal dose limit (Supplementary Fig. 6), the patient transitioned to a regimen of Abiraterone and Olaparib. Following a two-year hiatus from actinium-lutetium therapy, the tumor board reassessed the case (Fig. 3D). Given the persistence of bone metastases and the patient’s ineligibility for conventional therapies, tandem actinium-lutetium therapy was reinitiated. Prior to restarting treatment, significant genomic alterations occurred, resulting in a distinctly different tumor profile compared to the last assessment (Supplementary Fig. 7). Following the first cycle of reinitiated therapy, TFx increased from 0.41 to 0.61 accompanied by newly emerging genomic amplifications. In contrast, PSA levels remained relatively stable (82.1 ng/mL to 73.5 ng/mL).

### Patient 3 – mixed responder

**Fig. 4:**
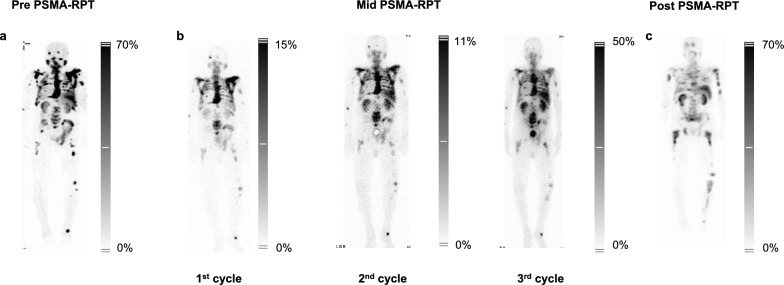
(**a**) Pre- and (**c**) post-treatment PSMA scintigraphy with [^99m^Tc]Tc-PSMA-GCK01 for Patient 3, treated with [^225^Ac]Ac-/[^177^Lu]Lu-PSMA-617. (**b**) Mid-treatment assessment refers to therapy-related scans conducted during the PSMA-RPT course

Patient 3 presented with extensive red marrow infiltration observed on PSMA-SPECT using [^99m^Tc]Tc-PSMA-GCK01 before starting PSMA-RPT (Fig. 4A). Actinium-lutetium tandem therapy initially led to a reduction in PSA levels, from over 446 ng/mL to 113 ng/mL, –74.6% (Supplementary Fig. 1.I). Similarly, TFx decreased from 0.15 to 0.04, reaching undetectable levels by the second cycle. However, during the third cycle, TFx rebounded to 0.15, signalling early disease progression. This rise in TFx (0.15 at the third cycle, Supplementary Fig. 1.J) preceded the PSA increase, highlighting its potential as an early biomarker for relapse detection. TFx further increased to 0.41 in the fourth cycle, confirming the relapse and mirroring baseline genomic patterns (Supplementary Fig. 8). Renal function remained stable, while hematologic monitoring showed fluctuating leukocyte counts and persistent mild to moderate anaemia (Supplementary Fig. 9).

### Patient 4 – non-responder

**Fig. 5:**
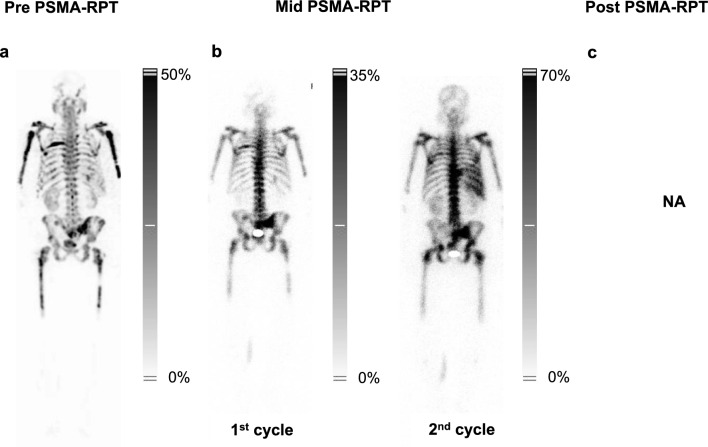
(**a**) Pre-therapy PSMA-SPECT/CT [^99m^Tc]Tc-PSMA-GCK01 was performed to evaluate baseline disease burden. (**b**) Mid-treatment scans were acquired following the first and second administration of [^225^Ac]Ac-/[^177^Lu]Lu-PSMA-617, to assess interim therapeutic response. (**c**) Post-therapy imaging was unavailable

Patient 4, presented with widespread liver and bone marrow lesions identified on a pre-treatment PSMA-SPECT scan using [^99m^Tc]Tc-PSMA-GCK01 (Fig. 5A). Actinium-lutetium tandem therapy initially reduced PSA levels by 37.6%, from 279 ng/mL to 174 ng/mL after the first cycle. However, by the second cycle, PSA levels surged by 686%, rising sharply to 1367 ng/mL, indicating rapid disease progression (Supplementary Fig. 1.M). TFx decreased from 0.68 to 0.48 after the first cycle, but increased to 0.82 by the second cycle (Supplementary Fig. 1.N). The CNA pattern remained stable and consistent with the baseline profile (Supplementary Fig. 10). Although white blood cell counts remained stable, the patient developed acute renal impairment, indicated by a sharp rise in creatinine and a drop in GFR (Supplementary Fig. 11).

## Discussion

The presented study investigates the potential of sequential ctDNA analysis as a biomarker for predicting treatment response, detecting resistance, and monitoring tumor evolution in mCRPC patients undergoing actinium-lutetium tandem therapy in a case study. We employed ULP-WGS of cfDNA, followed by ichorCNA, to determine the tumor load in cfDNA samples. The presented exploratory cases demonstrate the multifaceted utility of ctDNA in disease monitoring across four different PSMA-RPT trajectories. For instance, TFx levels in Patient 3 served as an early indicator of disease relapse, mirroring the metastatic landscape and signalling tumor resurgence (Supplementary Fig. 8). This capability to detect disease recurrence at a molecular level, preceding conventional biochemical markers or imaging, underscores the value of ctDNA as a prognostic tool as already reported for PSMA-RPT and other therapies [25–27]. Similarly, the synchronized reduction in TFx and PSA levels observed in Patient 1 reinforces the reliability of ctDNA as a surrogate marker for tumor burden, reflecting the systemic efficacy of PSMA-RPT (Supplementary Fig. 1.A and 1.B). In contrast, Patient 4, classified as a non-responder demonstrated persistent circulating tumor burden and a highly disrupted tumor genome (Supplementary Fig. 10), despite a transient reduction in TFx after the first treatment (Supplementary Fig. 1.N), the genomic landscape remained characterized by significant amplifications and deletions, highlighting the aggressiveness of the disease [28–30]. The comparative analysis of Patient 1 and Patient 4 particularly underscores the limitations of current biomarkers such as PSA, LDH, and ALP in reflecting disease dynamics. Both patients underwent complete prostatectomy but exhibited divergent PSA trajectories. Patient 1 displayed low PSA levels that did not accurately reflect the disease burden (Supplementary Fig. 1.A), while in Patient 4 elevated PSA levels (Supplementary Fig. 1.M) aligned more closely with their high tumor burden. Patient 2 underscores the value of ctDNA analysis in monitoring tumor burden at the genomic level, with PSA kinetics closely reflecting the decline in TFx, as previously reported [31, 32]. At reassessment, PSA levels suggested stable disease, but TFx increased markedly despite unchanged tumor volume on molecular imaging, indicating potential early signs of treatment resistance and a likely poor response to subsequent therapy cycles (Supplementary Fig. 7). These exploratory findings reiterate the variable reliability of PSA as a predictive biomarker for treatment response, as documented in the literature [32, 33]. Its variable reliability, particularly in cases like Patient 1, where PSA levels fail to accurately reflect the disease burden, creates significant challenges in clinical decision-making and monitoring [32, 34]. TFx could reflect changes in tumor dynamics more comprehensively; however, this observation is hypothesis-generating and would need to be explored further in larger patient cohorts with adequate statistical analyses [35]. A key challenge in managing mCRPC remains the detection of metastases, particularly micrometastases or lesions below the typical 3 mm detection limit. Patients may harbour tens or hundreds of micrometastases invisible to current imaging technologies. ULP-WGS also has limited sensitivity; in Patient 2, TFx was undetectable despite visible residual disease, emphasizing the trade-off between sequencing depth and cost. Metastases lacking PSMA expression, and therefore undetectable by addressing PSMA, pose a significant challenge to comprehensive disease assessment. This limitation highlights the value of ctDNA profiling as a real-time, minimally invasive approach capable of capturing tumor heterogeneity and detecting lesions missed by conventional imaging, thereby supporting more informed therapeutic decisions. While [^1^^8^F]FDG-PET can complement PSMA imaging by detecting metastases with low or absent PSMA expression–particularly those with high metabolic activity [36]–its routine integration is limited by factors such as scanner availability and healthcare infrastructure, especially in low-resource settings, rather than tracer availability itself [37]. ctDNA analysis could address this limitation by enabling the detection of both PSMA-positive and PSMA-negative lesions, thereby providing a more holistic view of the disease state in a cost-effective way. Although our exploratory findings are encouraging, they are derived from a small patient cohort and should therefore be interpreted with caution. An additional limitation is the absence of strict eligibility criteria, as all patients were treated under compassionate-use conditions. Consequently, the cohort is heterogeneous with respect to disease stage and prior therapies, making it challenging to fully disentangle biomarker dynamics from pre-treatment effects or intrinsic non-response. These factors limit the generalizability of our observations and underscore the need for larger, prospectively designed studies to validate the clinical utility of ctDNA monitoring in this setting. Despite these limitations, the nuanced molecular insights provided by ctDNA–such as the identification of actionable mutations and resistance mechanisms–hold significant promise for guiding patient selection and optimizing treatment strategies [38–42]. By leveraging ctDNA analysis, we can better identify patients who are most likely to benefit from PSMA-RPT with ^225^Ac, ensuring that this precious therapeutic resource is allocated effectively.

## Conclusion

This approach not only enhances treatment efficacy but also aligns with the principles of personalized medicine, paving the way for more precise and impactful cancer care. Our case study highlights the potential of ctDNA as a transformative biomarker to complement existing prognostic tools. The integration of ctDNA analysis into clinical practice could enable deeper insights into the tumor copy-number profile landscape, facilitating precise treatment adjustments and improved outcomes in mCRPC, particularly for actinium-lutetium tandem therapy. Beyond prostate cancer, ctDNA holds promise for understanding tumor evolution and resistance, paving the way for broader applications in personalized medicine and emerging therapies. Evaluation in larger cohorts is warranted.

## Supplementary Information

Additional file 1.

## Data Availability

All data analysed in this case study are presented within the article. Data availability is subject to local ethics committee approval. Reasonable requests for data should be directed to the corresponding author.
