# Supplementary material for: Exploratory case series of circulating tumor DNA dynamics during tandem therapy in metastatic castration-resistant prostate cancer
Source: EJNMMI Res. 2026 Feb 11;16:43. doi: 10.1186/s13550-026-01388-x (PMC13000081; doi:10.1186/s13550-026-01388-x)
Supplement: Supplementary file 1 — Additional file 1. [file 13550_2026_1388_MOESM1_ESM.docx]

**Exploratory Case Series of Circulating Tumor DNA Dynamics During Tandem Therapy in** **Metastatic Castration-Resistant Prostate Cancer**

**Authors:** Mariam Amghar^1,*^, Tobias Rausch^2^, Hilal Ozgur^2^, Mareike Roscher^3^, Ulrike Bauder-Wüst^1^, Frank Bruchertseifer^4^, Alfred Morgenstern^4^, Vladimír Beneš^2^, Clemens Kratochwil^5^, Martina Benešová-Schäfer^1,**^

**Affiliations:** Junior Research Group Translational Radiotheranostics^1^, Service Unit Radiopharmaceuticals und Preclinical Studies^3^, German Cancer Research Center (DKFZ), Heidelberg, Germany^1,3^, Genomics Core Facility, European Molecular Biology Laboratory (EMBL), Heidelberg, Germany^2^, European Commission, Joint Research Centre (JRC), Karlsruhe, Germany^4^, Department of Nuclear Medicine, University Hospital Heidelberg (UKHD), Heidelberg, Germany^5^.

**E-Mail addresses:**

[mariam.amghar@dkfz-heidelberg.de](mailto:mariam.amghar@dkfz-heidelberg.de); [rausch@embl.de](mailto:rausch@embl.de); [hilal.ozgur@embl.de](mailto:hilal.ozgur@embl.de); [mareike.roscher@dkfz-heidelberg.de](mailto:mareike.roscher@dkfz-heidelberg.de); [u.bauder-wuest@dkfz-heidelberg.de](mailto:u.bauder-wuest@dkfz-heidelberg.de); [frank.bruchertseifer@ec.europa.eu](mailto:frank.bruchertseifer@ec.europa.eu); [alfred.morgenstern@ec.europa.eu](mailto:alfred.morgenstern@ec.europa.eu); [benes@embl.de](mailto:benes@embl.de); [clemens.kratochwil@med.uni-heidelberg.de](mailto:clemens.kratochwil@med.uni-heidelberg.de); [m.benesova@dkfz-heidelberg.de](mailto:m.benesova@dkfz-heidelberg.de).

***First Author Contact Information:**

Dr. Mariam Amghar

German Cancer Research Center (DKFZ)

Im Neuenheimer Feld 280

69120 Heidelberg

Germany

**E-mail**: [mariam.amghar@dkfz-heidelberg.de](mailto:mariam.amghar@dkfz-heidelberg.de)

**Phone:** +49-6221-42-2690

**Fax:** +49-6221-42-5356

****Correspondence to:**

Dr. Martina Benešová-Schäfer

German Cancer Research Center (DKFZ)

Im Neuenheimer Feld 280

69120 Heidelberg

Germany

**E-mail**: [m.benesova@dkfz-heidelberg.de](mailto:m.benesova@dkfz-heidelberg.de)

**Phone:** +49-6221-42-5355

**Fax:** +49-6221-42-5356

**Supplementary data:**

**Table S1.** Overview of sequencing metrics for all samples, including error rate, median read length, duplicate fraction, and insert size.

| id | error_rate | median_read_length | duplicate_fraction | insert_size | coverage |
| --- | --- | --- | --- | --- | --- |
| Patient_2_baseline_1 | 0.004 | 110:110 | 0.156 | 175 | 1.29 |
| Patient_2_cycle1_2 | 0.003 | 111:111 | 0.147 | 182 | 1.81 |
| Patient_4_cycle1 | 0.003 | 111:111 | 0.130 | 171 | 1.83 |
| Patient_3_baseline | 0.003 | 111:111 | 0.182 | 179 | 2.11 |
| Patient_4_cycle3 | 0.004 | 111:111 | 0.168 | 166 | 2.40 |
| Patient_3_cycle1 | 0.003 | 111:111 | 0.174 | 180 | 0.64 |
| Patient_3_cycle2 | 0.003 | 111:111 | 0.179 | 174 | 2.46 |
| Patient_3_cycle3 | 0.004 | 111:111 | 0.180 | 182 | 1.80 |
| Patient_3_cycle4 | 0.004 | 111:111 | 0.151 | 189 | 1.71 |
| Patient_4_cycle2 | 0.004 | 111:111 | 0.107 | 176 | 1.08 |
| Patient_2_baseline | 0.004 | 111:111 | 0.151 | 175 | 1.27 |
| Patient_2_cycle1 | 0.003 | 111:111 | 0.122 | 166 | 0.35 |
| Patient_2_cycle2 | 0.004 | 111:111 | 0.124 | 180 | 0.87 |
| Patient_2_cycle3 | 0.003 | 111:111 | 0.122 | 168 | 1.02 |
| Patient_2_cycle4 | 0.004 | 111:111 | 0.113 | 206 | 7.44 |
| Patient_1_baseline | 0.004 | 111:111 | 0.164 | 176 | 2.00 |
| Patient_1_cycle1 | 0.003 | 111:111 | 0.130 | 168 | 1.10 |
| Patient_1_cycle2 | 0.003 | 111:111 | 0.126 | 169 | 0.74 |

**Table S2.** Overview of initial diagnostic data for the presented prostate cancer patients, including patient ID, initial diagnosis (iDiagnosis), initial Gleason Score (iGS), initial Prostate-specific Antigen (iPSA) levels, and initial tumor status. pT: Pathological / Clinical Tumor stage – extent of primary tumor based on pathology or imaging. pN / cN / N: Pathological / Clinical lymph node involvement. M / cM: Metastasis – clinical or confirmed distant spread of cancer. R0: Surgical margin status – R0 indicates no residual tumor (negative margins).

|  | iDiagnosis | iGS | iPSA (ng/mL) | iTumor Status |
| --- | --- | --- | --- | --- |
| Patient 1 | 2012 | 7b (4 + 3) | 19 | pT3b, pN0, R0 |
| Patient 2 | 2015 | 9 (4+5) | 60 | pT3b, pN1 |
| Patient 3 | 2015 | 7a (3+4) | 15 | pT1a, cN0, cM1 |
| Patient 4 | 2018 | 10 (5+5) | 32 | pT4, N1, M1 |


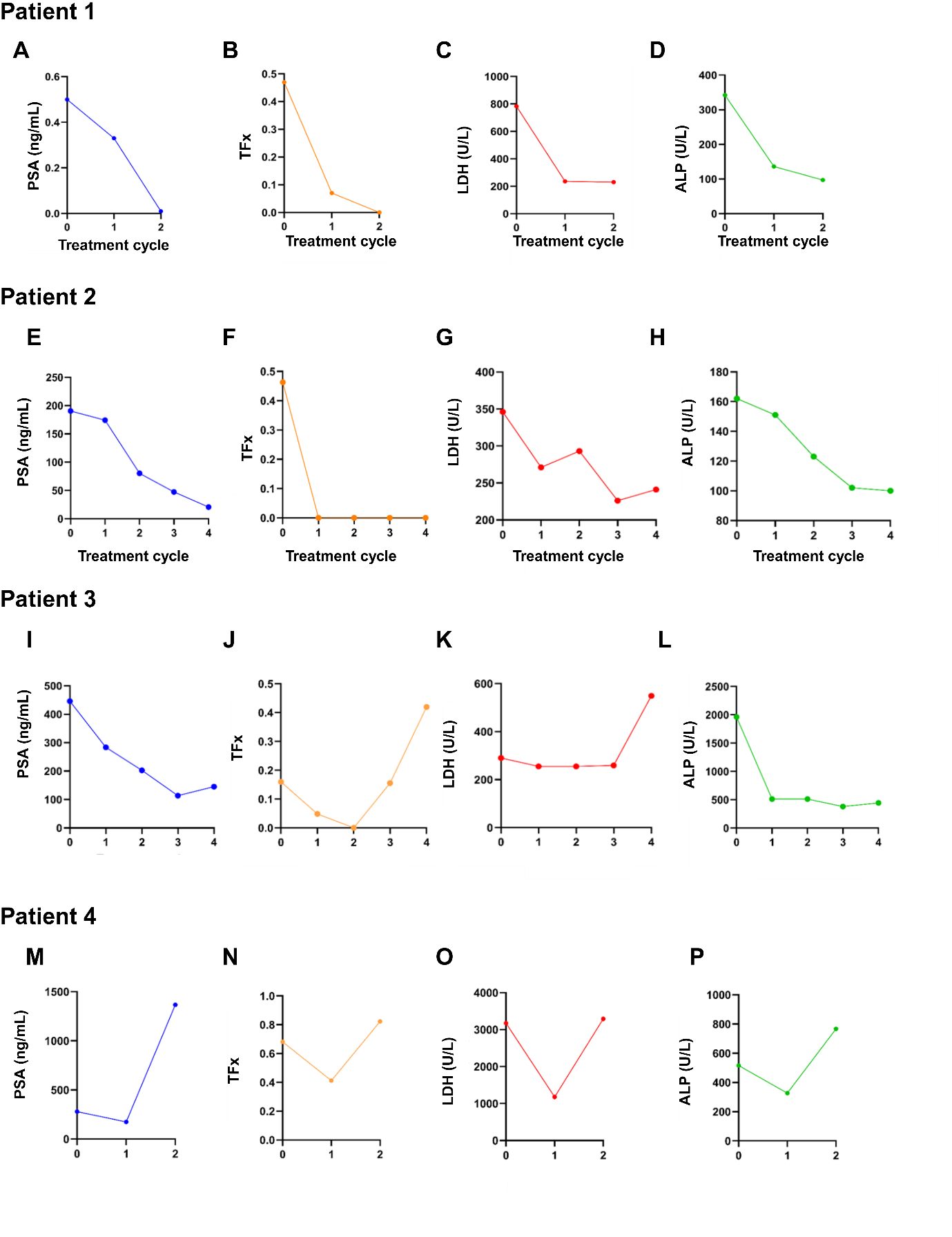
**Figure S1.** Representative patient-level trajectories of PSA, TFx, LDH, and ALP. Each row depicts data from an individual patient receiving [^225^Ac]Ac-/[^177^Lu]Lu-PSMA-617, with panels labelled alphabetically from left to right: (A–D) Patient 1, (E–H) Patient 2, (I–L) Patient 3, and (M–P) Patient 4. In Patient 1, biomarker trends reflect complete remission and a positive therapeutic response over two actinium-lutetium cycles. Patient 2 shows an initial response similar to Patient 1, characterized by parallel kinetics between PSA and TFx, accompanied by fluctuations in LDH levels during the second cycle. In Patient 3, PSA increases by the fourth cycle, indicating relapse; this relapse is preceded by an increase in TFx during the third cycle, signalling early disease progression, with a further increase by the fourth cycle. Patient 4 demonstrates minimal initial biomarker reduction, followed by elevations in all biomarkers, indicative of non-responsiveness to therapy. Abbreviations: PSA, prostate-specific antigen; TFx, tumor fraction; LDH, lactate dehydrogenase; ALP, alkaline phosphatase. Reference ranges: PSA < 4 ng/mL; TFx > 0.10 (indicative of ctDNA presence); LDH < 342 U/L; ALP 40–130 U/L.

**
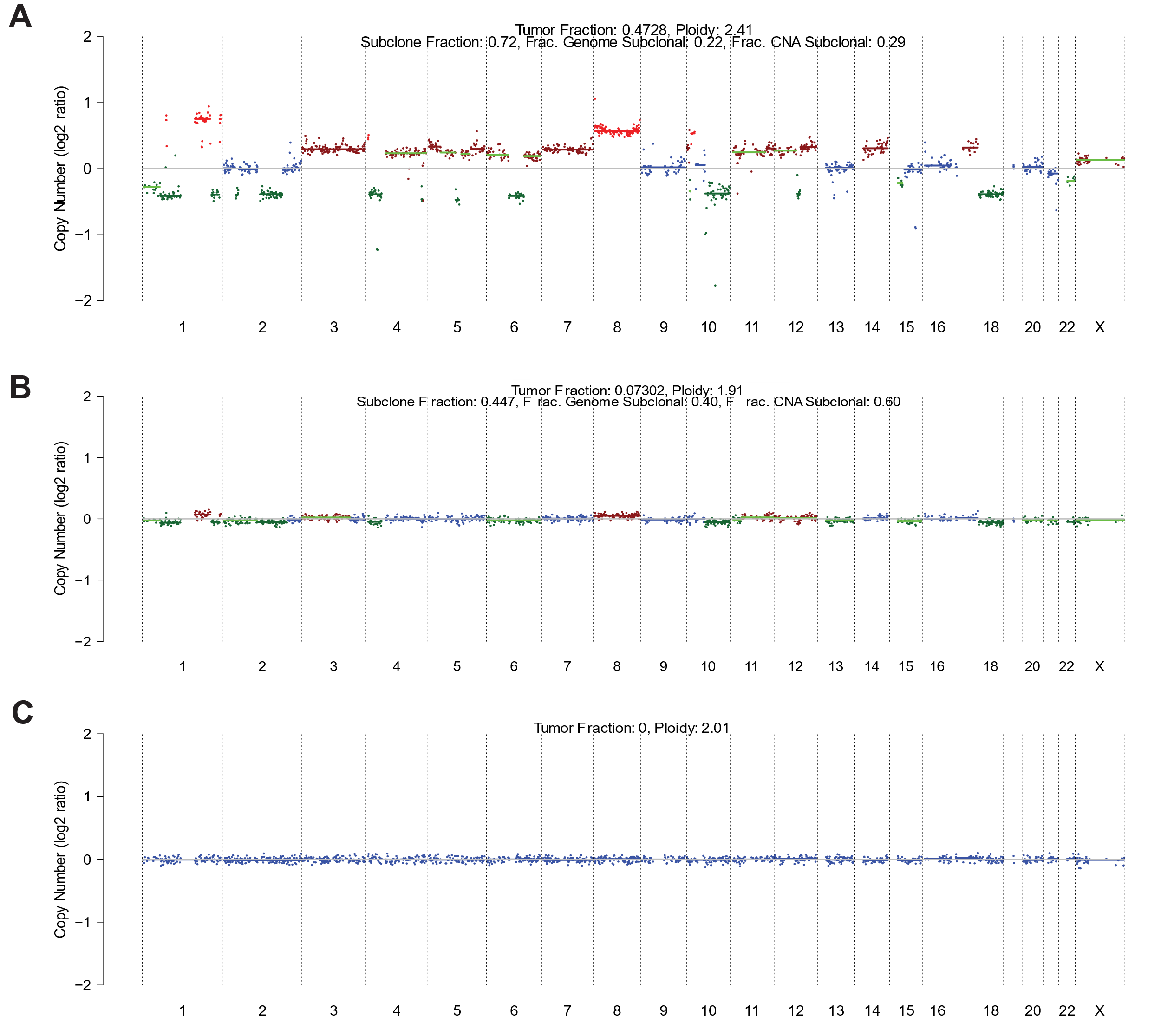
**

**Figure S2.** CNA profiles for Patient 1 across multiple treatment timepoints. Each panel represents a distinct timepoint: **A)** corresponds to the **baseline (pre-treatment)**, while **B), C),** represent the **first, second, third post-treatment cycles**, respectively. CNV profiles are depicted as log2 copy number ratios plotted against genomic coordinates.

**
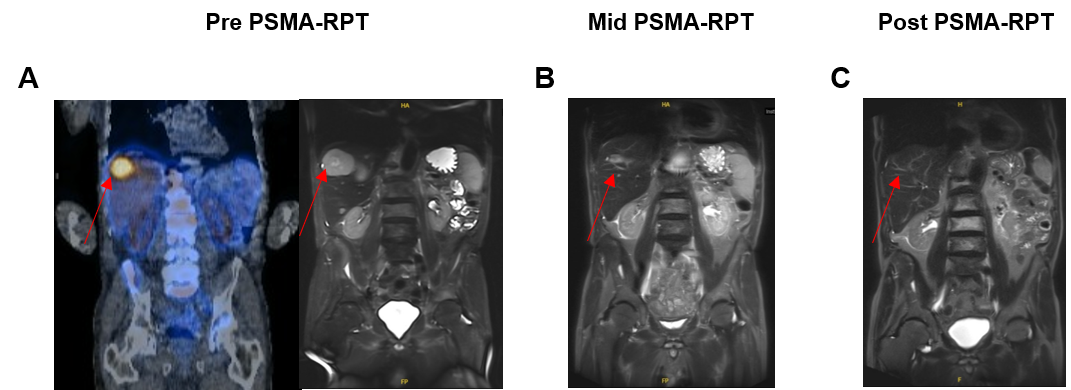
**

**Figure S3.** (A-B) Overview image sequence for Patient 1. (A) Liver metastasis (arrow) demonstrating intense uptake of [^99m^Tc]Tc-PSMA-GCK01 (A, left) and MRI (A, right) before treatment with [^225^Ac]Ac-/[^177^Lu]Lu-PSMA-617. Intermediate MRI revealing partial remission of this metastasis (B), and final MRI post-therapy (C), showcasing successful treatment impact.


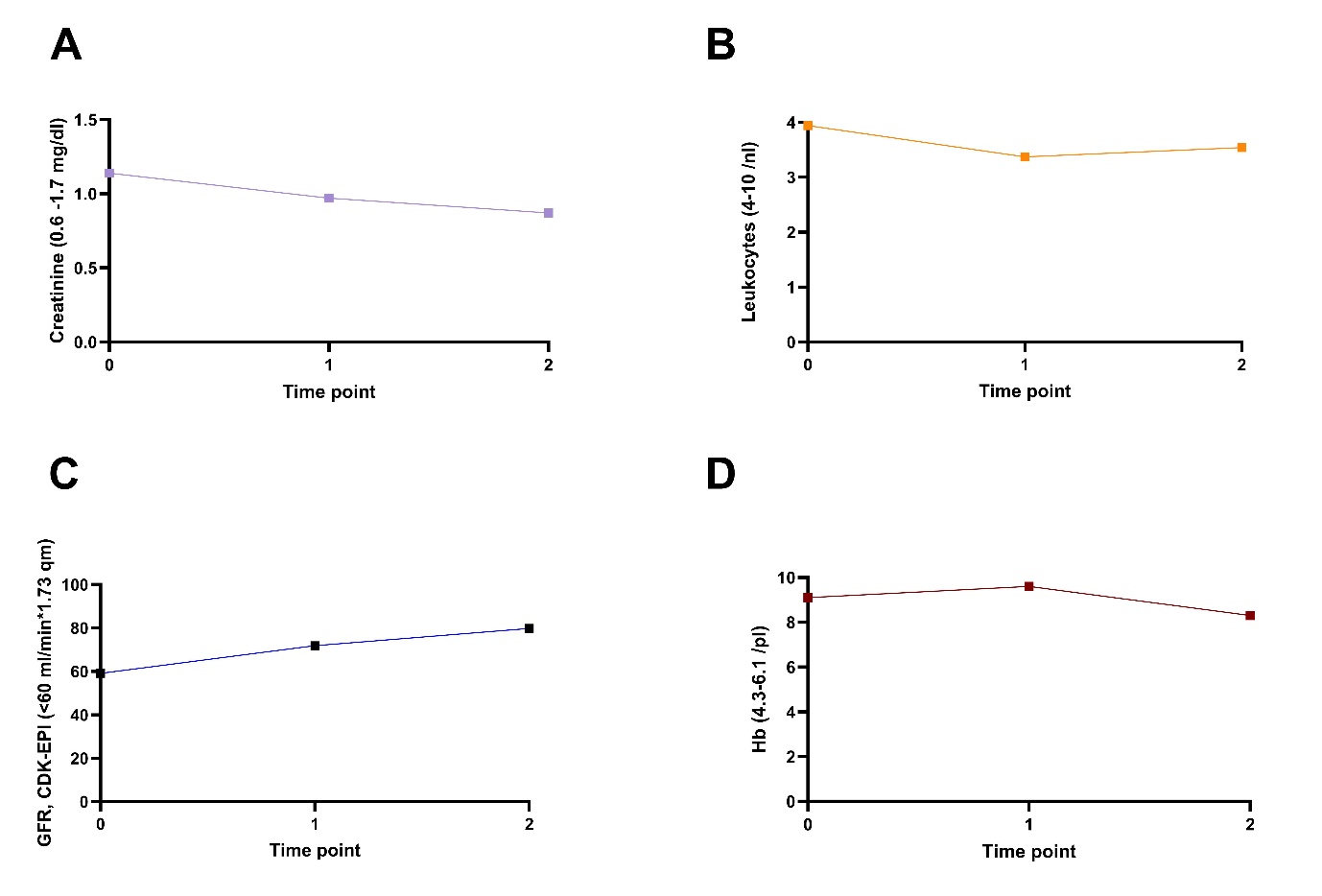


**Figure S4.** Charts illustrating the monitoring of Patient 1 at baseline and throughout treatment with [^225^Ac]Ac-/[^177^Lu]Lu-PSMA-617. These charts display the levels of: A) creatinine, B) leukocytes, C) glomerular filtration rate (GFR), calculated using the CDK-EPI formula, D) hemoglobin (Hb). The time points reflect biomarker kinetics over two cycles of RPT. Time point 0 represents baseline measurements, followed by assessments after the first treatment cycle and at the end of the second cycle.

*
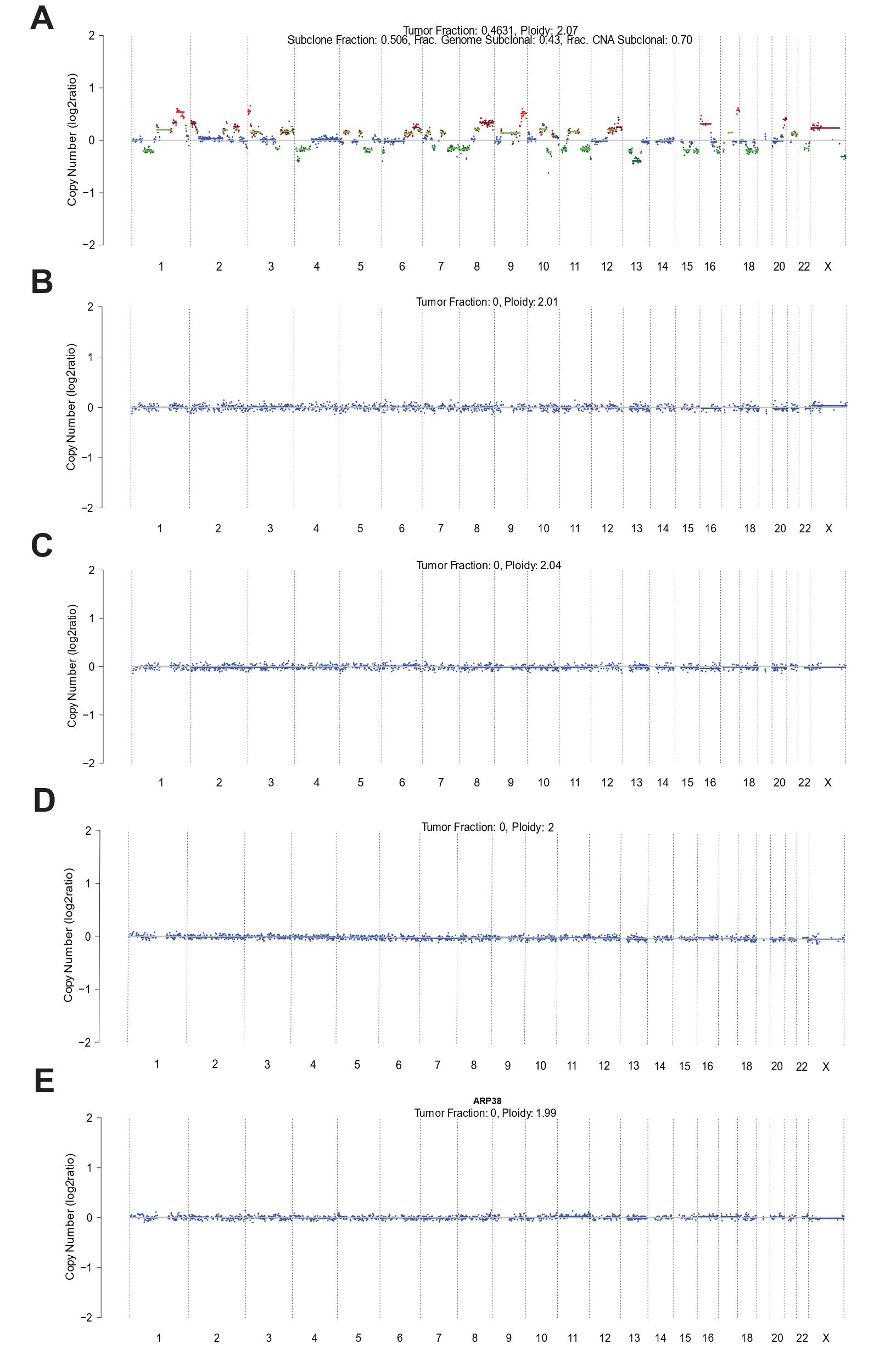
*

**Figure S5.** CNA profiles for Patient 2 across four treatment timepoints. Each panel represents a distinct timepoint: **A)** corresponds to the **baseline (pre-treatment)**, while **B), C), D),** and **E**) represent the **first, second, third, and fourth post-treatment cycles**, respectively. CNA profiles are presented as log2 copy number ratios (y-axis) across genomic coordinates (chromosomes 1 to X, x-axis). Colours indicate copy-number status: blue (neutral), brown (gain), green (deletion), and red (amplification). The plots illustrate a progressive reduction in TFx and CNAs over time, eventually falling below the detection limit. On the top legend the TFx is associated to each CNA profile.


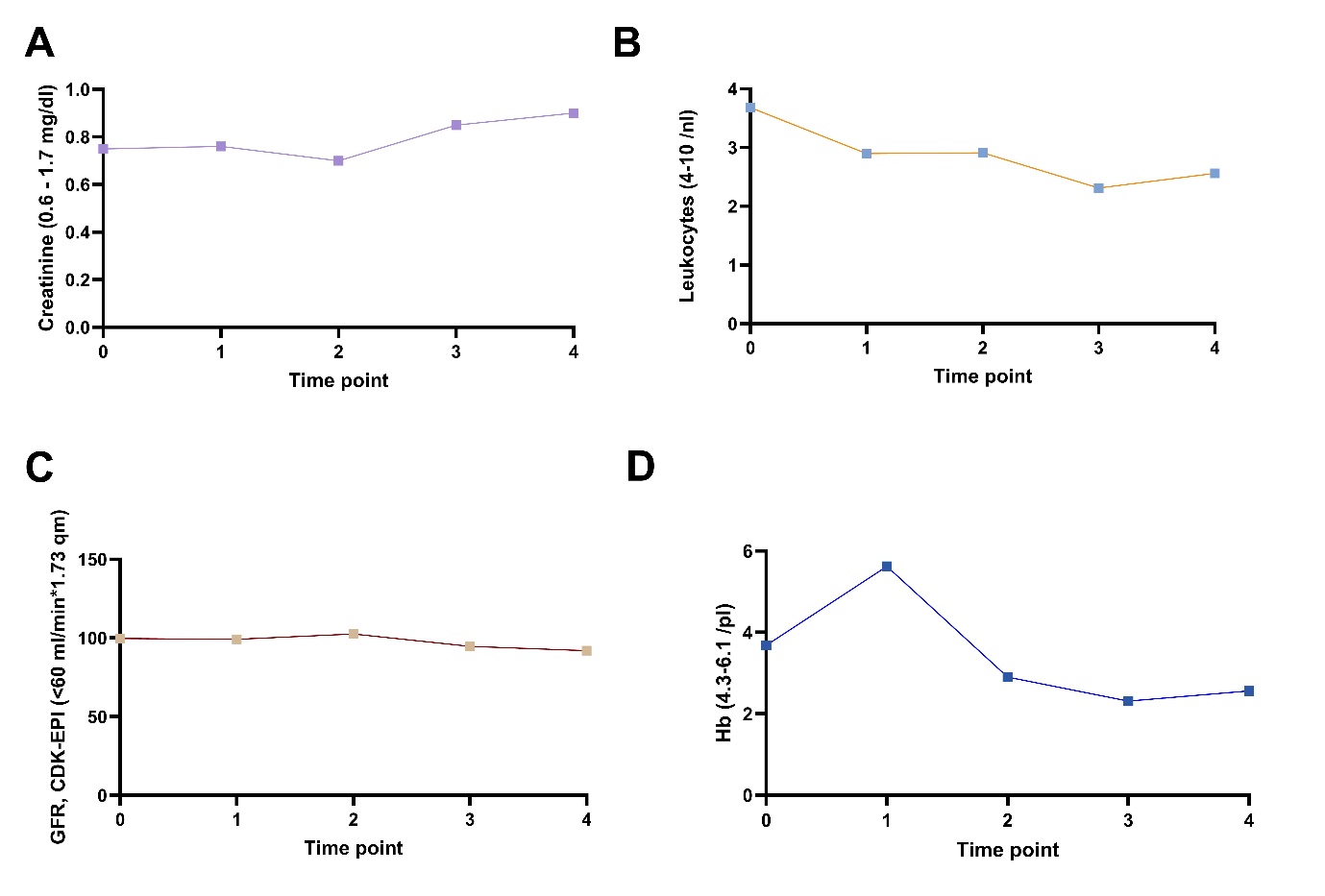
**Figure S6.** Charts illustrating the monitoring of Patient 2 at baseline and throughout treatment with [^225^Ac]Ac-/[^177^Lu]Lu-PSMA-617. These charts display the levels of: A) creatinine, B) leukocytes, C) glomerular filtration rate (GFR), calculated using the CDK-EPI formula, D) hemoglobin (Hb). The time points reflect biomarker kinetics over four cycles of RPT. Time point 0 represents baseline measurements, followed by assessments at each cycle of RPT therapy.


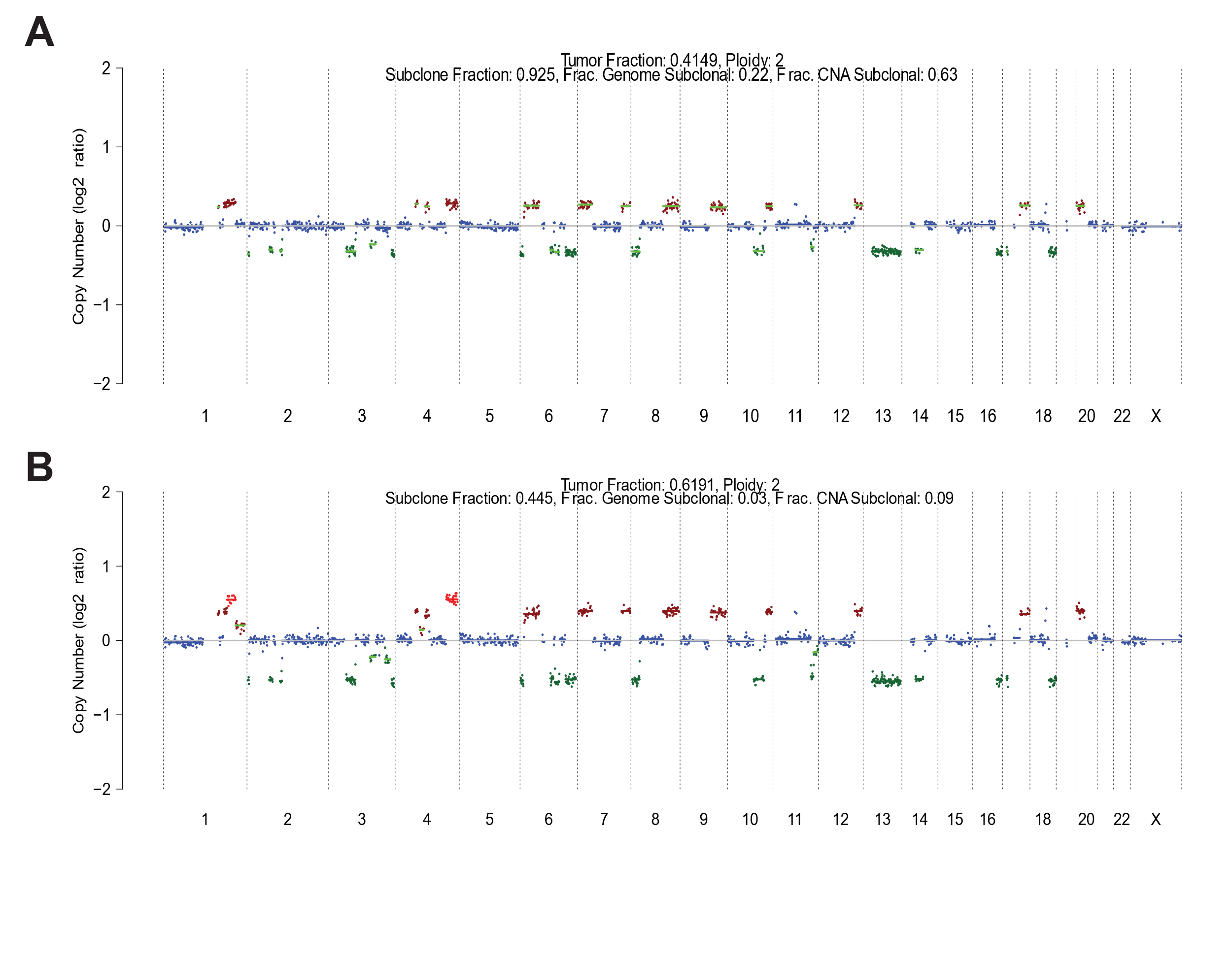
**Figure S7.** CNA profiles for Patient 2 for one treatment cycle. The upper panel A) shows the copy number alteration (CNA) profile at re-baseline, obtained after a two-year treatment break and prior to initiating a new series of therapy with [^225^Ac]Ac-/[^177^Lu]Lu-PSMA-617, following previous treatment with Olaparib and Abiraterone. The lower panel B) depicts the CNA profile after one treatment cycle. CNA profiles are presented as log2 copy number ratios (y-axis) across genomic coordinates (chromosomes 1 to X, x-axis). Colors indicate copy-number status: blue (neutral), brown (gain), green (deletion), and red (amplification). The plots illustrate a progressive reduction in TFx and CNAs over time, eventually falling below the detection limit. On the top legend the TFx is associated to each CNA profile. Subsequent panels display the CNA profiles after receiving one cycle of [^225^Ac]Ac-/[^177^Lu]Lu-PSMA-617. At the second baseline, the tumor profile had undergone significant changes, presenting a completely different genomic landscape compared to the initial assessment.


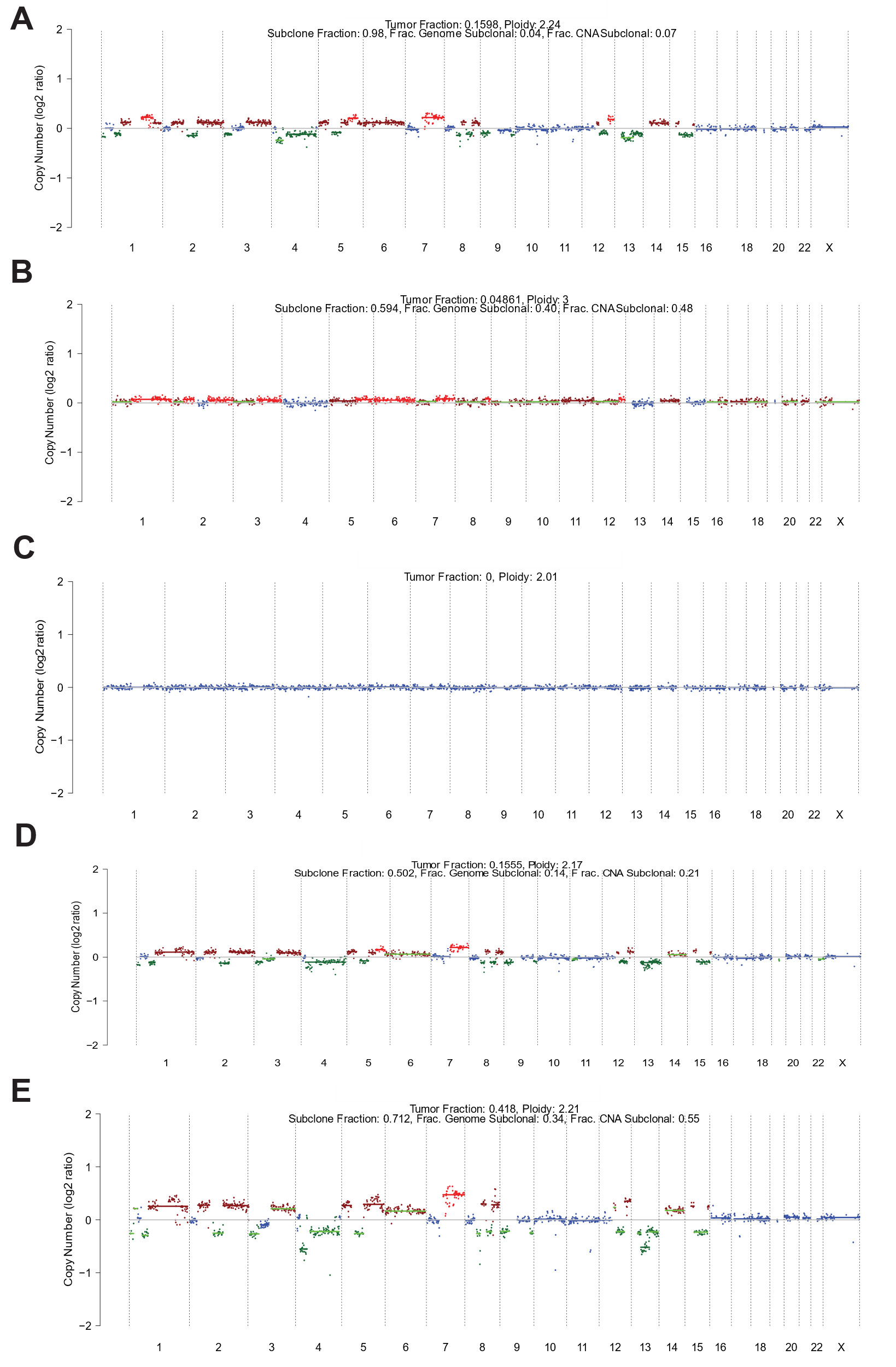
**Figure S8.** CNA profiles for Patient 3 across four treatment timepoints. Each panel represents a distinct timepoint: **A)** corresponds to the **baseline (pre-treatment)**, while **B), C), D),** and **E**) represent the **first, second, third, and fourth post-treatment cycles**, respectively. CNA profiles are presented as log2 copy number ratios (y-axis) across genomic coordinates (chromosomes 1 to X, x-axis). Colours indicate copy-number status: blue (neutral), brown (gain), green (deletion), and red (amplification). The plots illustrate a progressive reduction in TFx and CNAs over time, eventually falling below the detection limit. On the top legend the TFx is associated to each CNA profile. A positive therapeutic response is evident until the second cycle. However, by the third cycle, an increase in TFx and the re-emergence of CNA patterns resembling the baseline profile indicate early disease progression, which persists in the subsequent timepoint.


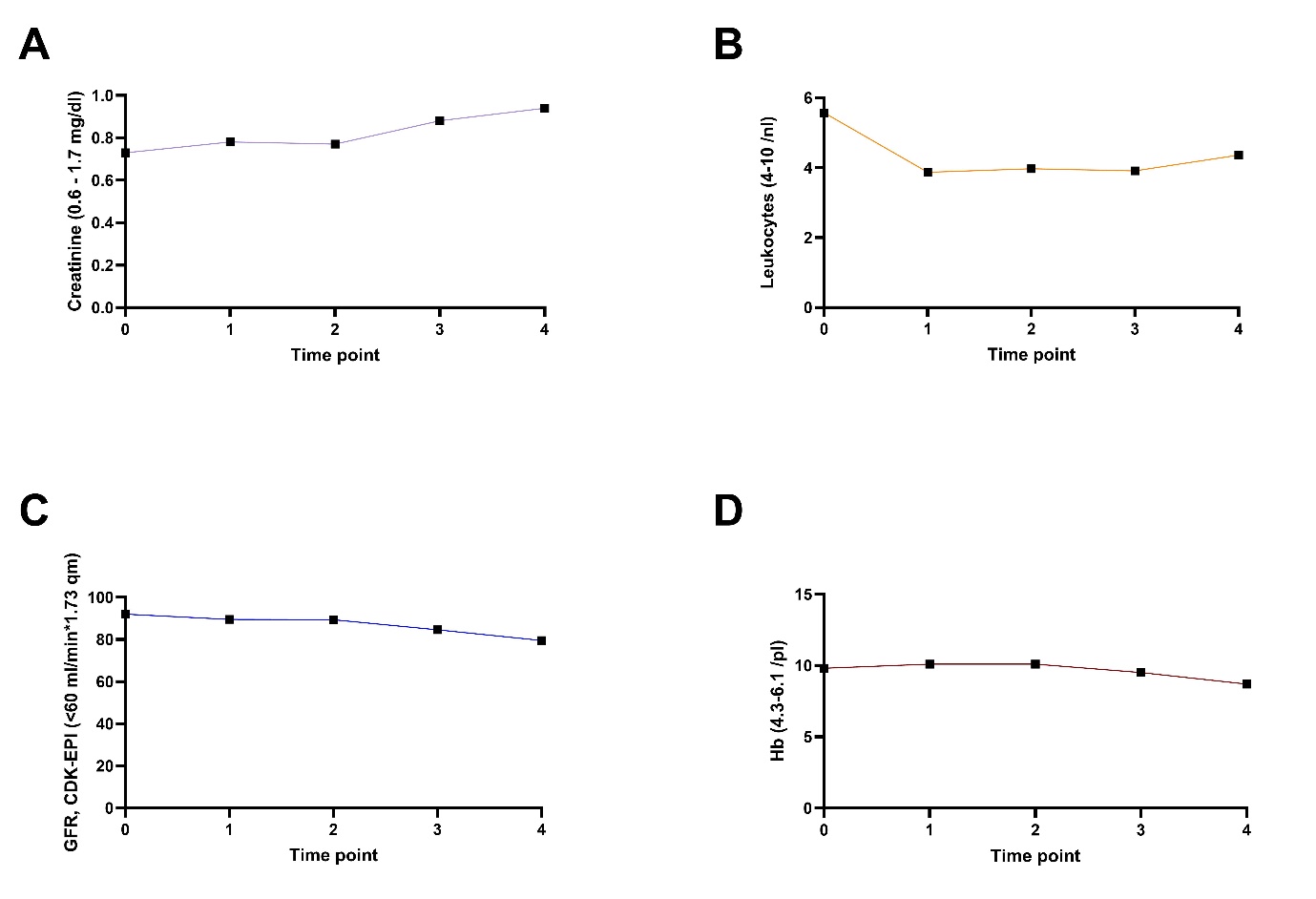


**Figure S9.** Charts illustrating the monitoring of Patient 3 at baseline and throughout treatment with [^225^Ac]Ac-/[^177^Lu]Lu-PSMA-617. These charts display the levels of: A) creatinine, B) leukocytes, C) glomerular filtration rate (GFR), calculated using the CDK-EPI formula, D) hemoglobin (Hb). The time points reflect biomarker kinetics over four cycles of RPT. Time point 0 represents baseline measurements, followed by assessments at each cycle of RPT therapy.


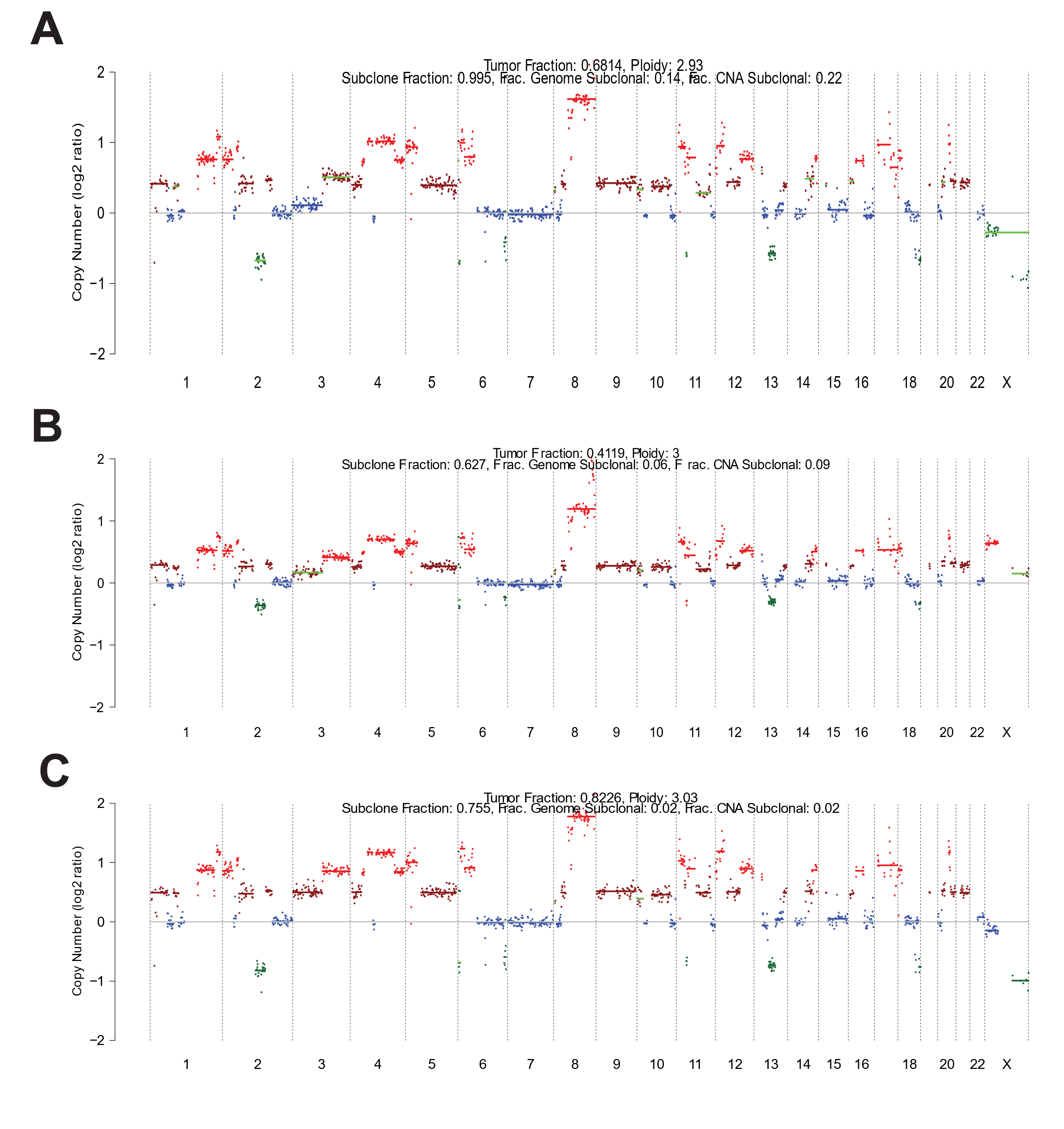


**Figure S10.** CNA profiles for Patient 4 across four treatment timepoints. Each panel represents a distinct timepoint, starting with the A) baseline (pre-treatment) followed by B), C) sequential post-treatment cycles. CNA profiles are presented as log2 copy number ratios (y-axis) across genomic coordinates (chromosomes 1 to X, x-axis). Colours indicate copy-number status: blue (neutral), brown (gain), green (deletion), and red (amplification). In the top legend, TFx is linked to each CNA profile. The plots depict a slight decrease in TFx, while genomic amplifications and deletions remain at a consistent magnitude.


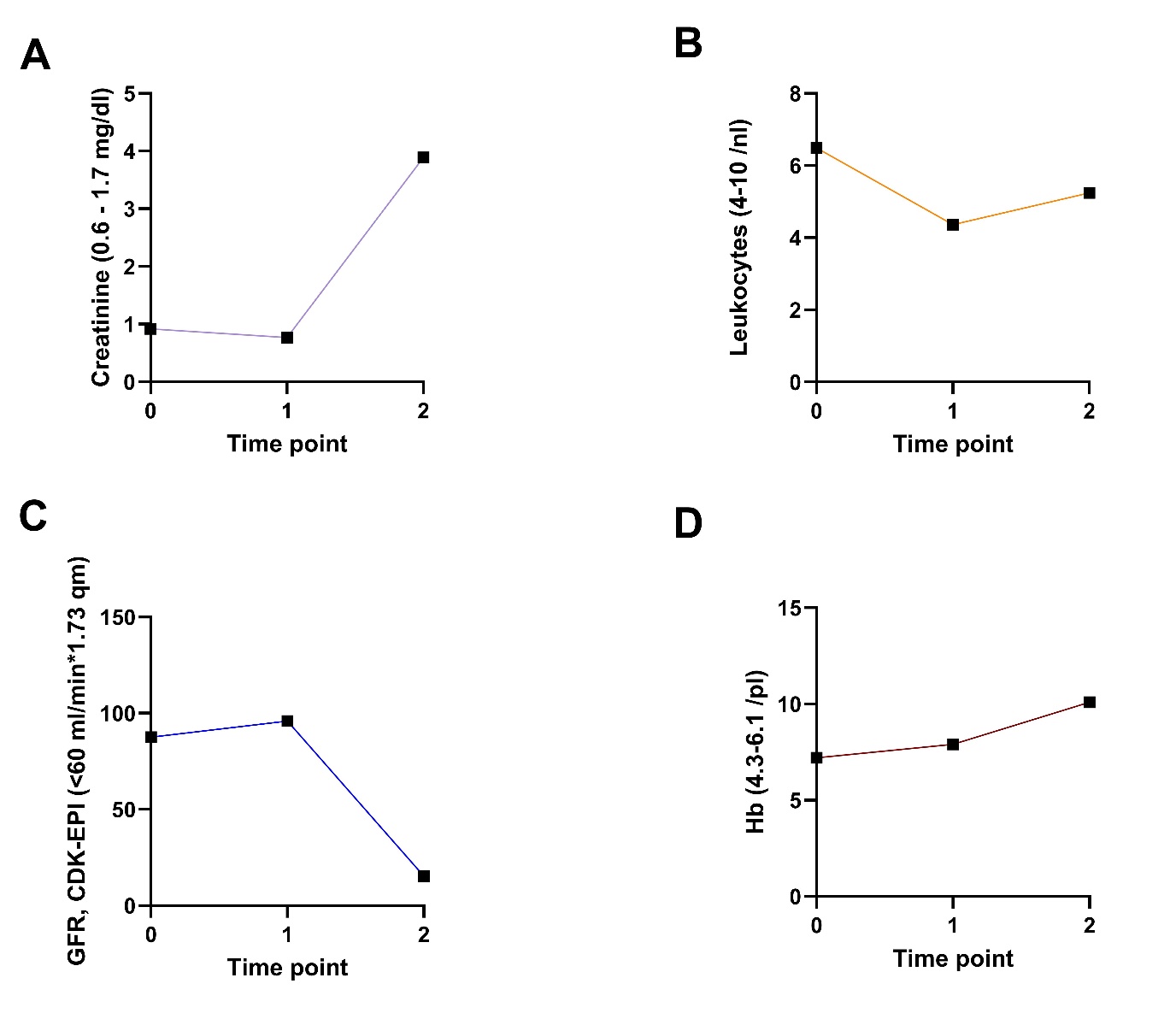


**Figure S11.** Charts illustrating the monitoring of Patient 4 at baseline and throughout treatment with [^225^Ac]Ac-/[^177^Lu]Lu-PSMA-617. These charts display the levels of: A) creatinine, B) leukocytes, C) glomerular filtration rate (GFR), calculated using the CDK-EPI formula, D) hemoglobin (Hb). The time points reflect biomarker kinetics over two cycles of RPT. Time point 0 represents baseline measurements, followed by assessments at each cycle of RPT therapy.
